# Supplementary material for: Identification of photoperiod-induced specific miRNAs in the adrenal glands of Sunite sheep (Ovis aries)
Source: Front Vet Sci. 2022 Jul 22;9:888207. doi: 10.3389/fvets.2022.888207 (PMC9354845; doi:10.3389/fvets.2022.888207)
Supplement: Supplementary file 1 [file Data_Sheet_1.ZIP › Supplementary file/miRDeep2 file.zip!/miRDeep2 file/Know_Structure/LP42A1/oar-mir-29b-2.pdf]

miRBase precursor : oar-mir-29b-2
Total read count : 12024
oar-miR-29b read count : 11080
remaining reads : 944

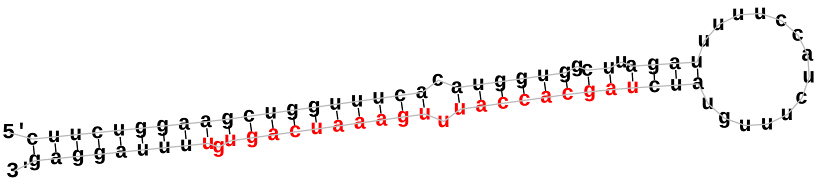

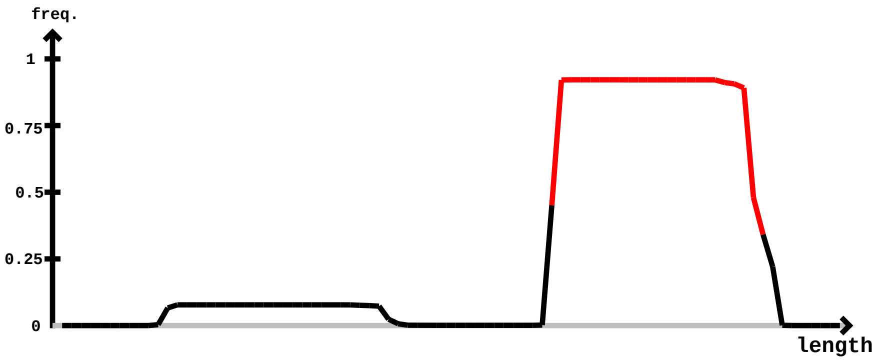


```
                                                   oar-miR-29b
5'- cuucuggaagcugguuucacaugguggcuuagauuuuuccaucuuuguaucuagcaccauuugaaaucaguguuuuaggag   -3'    exp
    (((((((((((((((((((.((((((.((.((((..............)))))))))))).)))))))))).)))))))))    reads      mm         sample
    .........gcugguuucacaugguggcuu...................................................    2          0          seq
    .........gcugguuucacaugguggcuua..................................................    2          0          seq
    .........gcugguuucacaugguggcuuag.................................................    4          0          seq
    .........gcugguuucacaugguggcuuaga................................................    34         0          seq
    ..........cugguuucacaugguggcuu...................................................    16         0          seq
    ..........cugguuucacaugguggcuua..................................................    13         0          seq
    ..........cugguuucacaugguggcuuag.................................................    16         0          seq
    ..........cugguuucacaugguggcuuaga................................................    496        0          seq
    ..........cugguuucacaugguggcuuagau...............................................    159        0          seq
    ..........cugguuucacaugguggcuuagauu..............................................    49         0          seq
    ..........cugguuucacaugguggcuuagauuu.............................................    3          0          seq
    ..........cugguuucacaugguggcuuagauuuu............................................    1          0          seq
    ...........ugguuucacaugguggcu....................................................    1          0          seq
    ...........ugguuucacaugguggcuuag.................................................    4          0          seq
    ...........ugguuucacaugguggcuuaga................................................    72         0          seq
    ...........ugguuucacaugguggcuuagau...............................................    45         0          seq
    ...........ugguuucacaugguggcuuagauu..............................................    13         0          seq
    ...........ugguuucacaugguggcuuagauuu.............................................    2          0          seq
    ...........ugguuucacaugguggcuuagauuuuu...........................................    1          0          seq
    .................................uuuuuccaucuuuguauc..............................    10         0          seq
    ....................................uuccaucuuuguaucuagcacc.......................    1          0          seq
    .................................................ucuagcaccauuugaaauc.............    1          0          seq
    .................................................ucuagcaccauuugaaaucGg...........    1          1          seq
    .................................................ucuagcaccauuugaaaucagu..........    10         0          seq
    ..................................................cuagcaccauuugaaau..............    2          0          seq
    ..................................................cuagcaccauuugaaauc.............    55         0          seq
    ..................................................cuagcaccauuugaaauca............    14         0          seq
    ..................................................cuagcaccauuugaaaucGg...........    1          1          seq
    ..................................................cuagcaccauuugaaaucag...........    22         0          seq
    ..................................................cuagcaccauuugaaaucagu..........    2921       0          seq
    ..................................................cuagcaccauuugaaaucGgu..........    31         1          seq
    ..................................................cuagcaccauuugaaaucagug.........    1286       0          seq
    ..................................................cuagcaccauuugaaaucagugu........    1069       0          seq
    ..................................................cuagcaccauuugaaaucaguguu.......    6          0          seq
    ...................................................uGgcaccauuugaaauc.............    1          1          seq
```

oar-miR-29b

```
cuucuggaagcugguuucacaugguggcuuagauuuuuccaucuuuguaucuagcaccauuugaaaucaguguuuuaggag
...................................................uagcaccauuugaaauU.............    6      1     seq
...................................................uagcaccauuugaaauA.............    1      1     seq
...................................................uagcaccauuugaaauc.............    58     0     seq
...................................................uagcaccauuugaaaucG............    29     1     seq
...................................................uagcaccauuugaaauca............    14     0     seq
...................................................uagcaccauuugaaaucag...........    44     0     seq
...................................................uagcaccauuugaaaucGg...........    115    1     seq
...................................................uagcaccauuugaaaucGgu..........    649    1     seq
...................................................uagcaccauuugaaaucagu..........    1333   0     seq
...................................................uagcaccauuugaaaucagug.........    379    0     seq
...................................................uagcaccauuugaaaucagugu........    384    0     seq
...................................................uagcaccauuugaaaucaguguu.......    2628   0     seq
...................................................uagcaccauuugaaaucaguguuu......    10     0     seq
...................................................uagcaccauuugaaaucaguguuC......    1      1     seq
...................................................uagcaccauuugaaaucaguguuuu.....    1      0     seq
....................................................agcaccauuugaaaucagu..........    2      0     seq
....................................................agcaccauuugaaaucGgu..........    1      1     seq
....................................................agcaccauuugaaaucaguguu.......    5      0     seq
```
